# Supplementary material for: Causal Relationship between Aging and Anorexia Nervosa: A White-Matter-Microstructure-Mediated Mendelian Randomization Analysis
Source: Biomedicines. 2024 Aug 16;12(8):1874. doi: 10.3390/biomedicines12081874 (PMC11351342; doi:10.3390/biomedicines12081874)
Supplement: Supplementary file 1 [file biomedicines-12-01874-s001.zip › Supplementary Figure/Figure S4/Singe SNP plot of MR analysis for TL to ubm-b-2100.pdf]

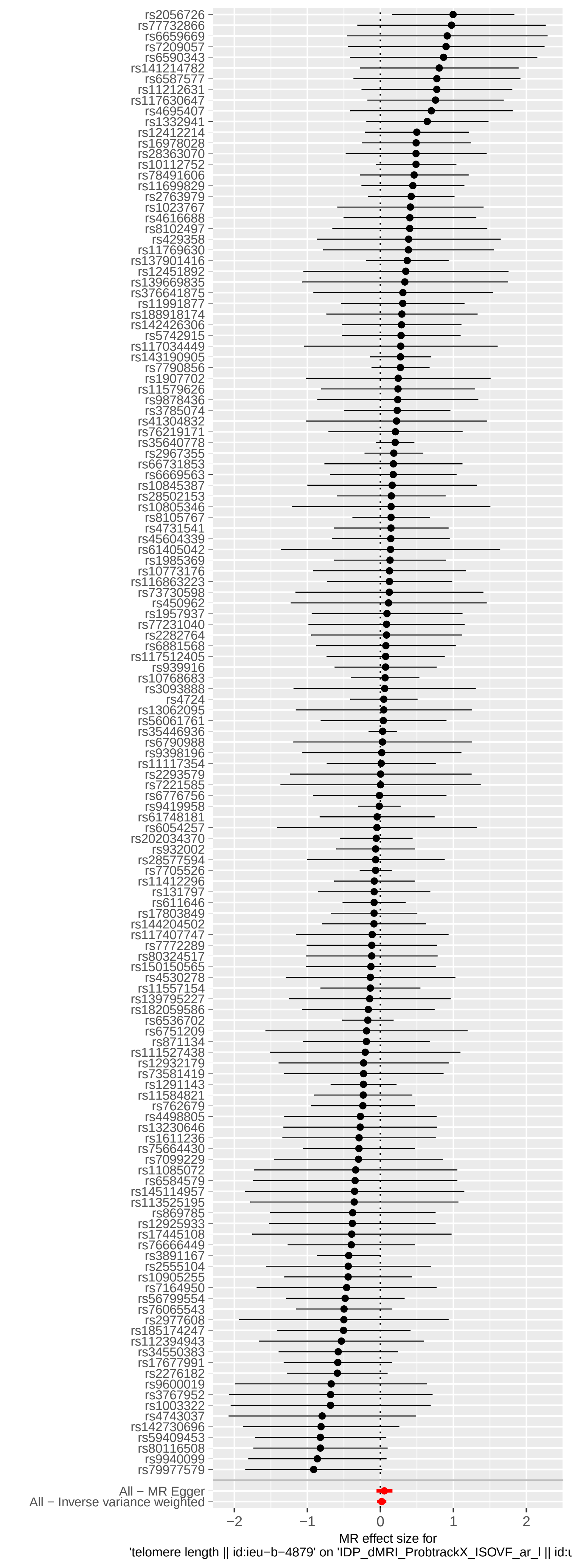

rs2056726
rs77732866
rs6659669
rs7209057
rs6590343
rs141214782
rs6587577
rs11212631
rs117630647
rs4695407
rs1332941
rs12412214
rs16978028
rs28363070
rs10112752
rs78491606
rs11699829
rs2763979
rs1023767
rs4616688
rs8102497
rs429358
rs11769630
rs137901416
rs12451892
rs139669835
rs376641875
rs11991877
rs188918174
rs142426306
rs5742915
rs117034449
rs143190905
rs7790856
rs1907702
rs11579626
rs9878436
rs3785074
rs41304832
rs76219171
rs35640778
rs2967355
rs66731853
rs6669563
rs10845387
rs28502153
rs10805346
rs8105767
rs4731541
rs45604339
rs61405042
rs1985369
rs10773176
rs116863223
rs73730598
rs450962
rs1957937
rs77231040
rs2282764
rs6881568
rs117512405
rs939916
rs10768683
rs3093888
rs4724
rs13062095
rs56061761
rs35446936
rs6790988
rs9398196
rs11117354
rs2293579
rs7221585
rs6776756
rs9419958
rs61748181
rs6054257
rs202034370
rs932002
rs28577594
rs7705526
rs11412296
rs131797
rs611646
rs17803849
rs144204502
rs117407747
rs7772289
rs80324517
rs150150565
rs4530278
rs11557154
rs139795227
rs182059586
rs6536702
rs6751209
rs871134
rs111527438
rs12932179
rs73581419
rs1291143
rs11584821
rs762679
rs4498805
rs13230646
rs1611236
rs75664430
rs7099229
rs11085072
rs6584579
rs145114957
rs113525195
rs869785
rs12925933
rs17445108
rs76666449
rs3891167
rs2555104
rs10905255
rs7164950
rs56799554
rs76065543
rs2977608
rs185174247
rs112394943
rs34550383
rs17677991
rs2276182
rs9600019
rs3767952
rs1003322
rs4743037
rs142730696
rs59409453
rs80116508
rs9940099
rs79977579
All − MR Egger
All − Inverse variance weighted
−2
−1
0
1
2
MR effect size for
'telomere length || id:ieu−b−4879' on 'IDP_dMRI_ProbtrackX_ISOVF_ar_l || id:u
